# Supplementary material for: Pareto rules for malaria super-spreaders and super-spreading
Source: Nat Commun. 2019 Sep 2;10:3939. doi: 10.1038/s41467-019-11861-y (PMC6718398; doi:10.1038/s41467-019-11861-y)
Supplement: Supplementary file 2 — Reporting Summary [file 41467_2019_11861_MOESM2_ESM.pdf]

nature research

Corresponding author(s): David L Smith

Last updated by author(s): Aug 1, 2019

# Reporting Summary

Nature Research wishes to improve the reproducibility of the work that we publish. This form provides structure for consistency and transparency in reporting. For further information on Nature Research policies, see Authors & Referees and the Editorial Policy Checklist.

## Statistics

For all statistical analyses, confirm that the following items are present in the figure legend, table legend, main text, or Methods section.

| n/a | Confirmed | |
|---|---|---|
| ☒ | ☐ | The exact sample size ($n$) for each experimental group/condition, given as a discrete number and unit of measurement |
| ☐ | ☒ | A statement on whether measurements were taken from distinct samples or whether the same sample was measured repeatedly |
| ☒ | ☐ | The statistical test(s) used AND whether they are one- or two-sided<br>*Only common tests should be described solely by name; describe more complex techniques in the Methods section.* |
| ☒ | ☐ | A description of all covariates tested |
| ☒ | ☐ | A description of any assumptions or corrections, such as tests of normality and adjustment for multiple comparisons |
| ☒ | ☐ | A full description of the statistical parameters including central tendency (e.g. means) or other basic estimates (e.g. regression coefficient) AND variation (e.g. standard deviation) or associated estimates of uncertainty (e.g. confidence intervals) |
| ☒ | ☐ | For null hypothesis testing, the test statistic (e.g. $F$, $t$, $r$) with confidence intervals, effect sizes, degrees of freedom and $P$ value noted<br>*Give P values as exact values whenever suitable.* |
| ☒ | ☐ | For Bayesian analysis, information on the choice of priors and Markov chain Monte Carlo settings |
| ☒ | ☐ | For hierarchical and complex designs, identification of the appropriate level for tests and full reporting of outcomes |
| ☒ | ☐ | Estimates of effect sizes (e.g. Cohen's $d$, Pearson's $r$), indicating how they were calculated |

*Our web collection on statistics for biologists contains articles on many of the points above.*

## Software and code

Policy information about availability of computer code

| | |
|---|---|
| Data collection | No software was used to collect these data. |
| Data analysis | All code used for these analyses was also used for a previous paper. It is publicly available online at [https://gatesopenresearch.s3.amazonaws.com/supplementary/12838/f7b1ea69-1fb5-46e5-98d8-5601c4d6f731.R]. The analysis for this paper, including the code to do the Pareto analysis and to generate the figures is available for download in a single file, called Figures.tgz from [https://figshare.com/s/b273374317531a2e4377]. The figures can be remade in R by executing the file Figures/MASTER-SCRIPT.R. Scripts to create each one of the individual figures is found in the subdirectory Figures/scripts. |

For manuscripts utilizing custom algorithms or software that are central to the research but not yet described in published literature, software must be made available to editors/reviewers. We strongly encourage code deposition in a community repository (e.g. GitHub). See the Nature Research guidelines for submitting code & software for further information.

## Data

Policy information about availability of data

All manuscripts must include a data availability statement. This statement should provide the following information, where applicable:

- Accession codes, unique identifiers, or web links for publicly available datasets
- A list of figures that have associated raw data
- A description of any restrictions on data availability

The primary data generated and analysed during the current study are available at figshare [https://doi.org/10.6084/m9.figshare.6797408.v3]. A table for each site containing a row for each observation: the count, the computed expected number of bites for that day, the biting weight for the individual is available for download (https://figshare.com/articles/Uganda_environmental_covariates_csv/6797408/3) .
Other data from the same study can be found in the Clinical Epidemiology Database Resources repository [https://clinepidb.org/ce/app]. Primary input and output of this analysis is also included in a downloadable file called Figures.tgz from [https://figshare.com/s/b273374317531a2e4377]. All these files are found in the

subdirectory Figures/inputs.

# Field-specific reporting

Please select the one below that is the best fit for your research. If you are not sure, read the appropriate sections before making your selection.

☐ Life sciences ☐ Behavioural & social sciences ☒ Ecological, evolutionary & environmental sciences

For a reference copy of the document with all sections, see nature.com/documents/nr-reporting-summary-flat.pdf

# Ecological, evolutionary & environmental sciences study design

All studies must disclose on these points even when the disclosure is negative.

| | |
|---|---|
| Study description | We selected 100 households randomly at three sites in Uganda. At each house, we sampled mosquitoes using CDC Light traps once a month. |
| Research sample | Prior to conducting the entomological surveys, all households within each subcounty were enumerated and mapped to generate a sampling frame for the random selection of houses representative of the catchment areas (Walukuba, Jinja 9,881 households; Kihihi, Kanungu 12,774 households; Nagongera, Tororo 6,992 households). |
| Sampling strategy | We selected 100 households randomly at each site. |
| Data collection | Light traps were positioned indoors next to a child aged six months to ten years, sleeping under a long-lasting insecticidal net (LLIN). We used miniature CDC light traps (Model 512; John W. Hock Company, Gainesville, FL, USA). The traps were positioned with the light bulb 1 m above the floor at the foot end of the bed where a person slept under a LLIN. Traps were set at 19.00 and collected at 07.00 the following morning. If it was not possible to set the trap in the intended house, it was moved to the nearest similar house.<br><br>P. falciparum sporozoites were identified in individual mosquitoes stored on desiccant using an ELISA technique. The P. falciparum sporozoite rate (PfSR) is the number of mosquitoes infected with sporozoites divided by the total number of mosquitoes examined using each respective method of mosquito collection, expressed as a percentage. A stopping rule was deployed, for the practical reason of limiting expenses, so that a maximum of 50 mosquitoes were tested from any trap in any site which caught more than this amount. |
| Timing and spatial scale | Collections were made monthly. Each night approximately 12 traps were set for four nights each week. They were rotated in the same order each month. |
| Data exclusions | If the occupant did not spend the night in the selected room or if the trap was faulty, the data were excluded from the analysis. |
| Reproducibility | The study method can be reproduced. |
| Randomization | We selected 100 households randomly at each site. |
| Blinding | It was not possible to conduct a blinded study. |

Did the study involve field work? ☒ Yes ☐ No

## Field work, collection and transport

| | |
|---|---|
| Field conditions | All sampling was done indoors. Light traps were positioned indoors next to a child aged six months to ten years, sleeping under a long-lasting insecticidal net (LLIN). |
| Location | Our study had three sites in Uganda: Walukuba, Jinja District (00° 26' 33.2" N, 33°13' 32.3" E); Kihihi, Kanungu District (00°45' 03.1" S, 29°42' 03.6" E) , and Nagongera, Tororo District (00°46' 10.6" , N 34°01' 34.1" E) . |
| Access and import/export | Mosquito samples were transported to Kampala, Uganda and stored at room temperature. |
| Disturbance | All sampling was done indoors. |

# Reporting for specific materials, systems and methods

We require information from authors about some types of materials, experimental systems and methods used in many studies. Here, indicate whether each material, system or method listed is relevant to your study. If you are not sure if a list item applies to your research, read the appropriate section before selecting a response.

## Materials & experimental systems

| n/a | Involved in the study |
|---|---|
| ☒ | ☐ Antibodies |
| ☒ | ☐ Eukaryotic cell lines |
| ☒ | ☐ Palaeontology |
| ☐ | ☒ Animals and other organisms |
| ☐ | ☒ Human research participants |
| ☒ | ☐ Clinical data |

## Methods

| n/a | Involved in the study |
|---|---|
| ☒ | ☐ ChIP-seq |
| ☒ | ☐ Flow cytometry |
| ☒ | ☐ MRI-based neuroimaging |

# Animals and other organisms

Policy information about studies involving animals; ARRIVE guidelines recommended for reporting animal research

| | |
|---|---|
| Laboratory animals | N/A |
| Wild animals | N/A |
| Field-collected samples | We caught mosquitoes in a CDC Light Trap. |
| Ethics oversight | N/A. Our study involved only mosquitoes. |

Note that full information on the approval of the study protocol must also be provided in the manuscript.

# Human research participants

Policy information about studies involving human research participants

| | |
|---|---|
| Population characteristics | We enrolled approximately 100 households at each site. Light traps were positioned indoors next to a child aged six months to ten years, sleeping under a long-lasting insecticidal net (LLIN). |
| Recruitment | Written informed consent from the head of household or an adult household representative was obtained by the field worker prior to conducting surveillance in a household. Field workers provided written informed consent for the human-landing catches, were paid for their work and provided with malaria chemoprophylaxis, consisting of mefloquine (250 mg tab orally once weekly) or doxycycline (100 mg tab orally each day). They were also offered medical treatment for any illness that developed during the period of their employment. |
| Ethics oversight | Ethical approval for this study was provided by the Uganda National Council for Science and Technology, the Makerere University School of Medicine Research and Ethics Committee, the University of California, San Francisco Committee on Human Research, London School of Hygiene and Tropical Medicine ethical committee and the School of Biological and Biomedical Sciences Ethics Committee, Durham University. |

Note that full information on the approval of the study protocol must also be provided in the manuscript.
